# Editorial Brevities

**Published:** 1888-03

**Authors:** 


### EDITORIALS AND MISCELLANEOUS.

### EDITORIAL BREVITIES.

SEE the new advertisement of Fellow's hypophosphites in this issue of our journal.

APPARATUS FOR DEFORMITIES.—See advertisement of Max Wocher & Son in regard to apparatus for deformities.

HAWLEY'S PEPSINE.—Physicians would do well to examine the advertisement of Hawley's pepsine, and give the article a test in practice.

SEE advertisement of National Surgical Institute, a private orthopædic hospital.

I. PHILLIPS, of Atlanta, the polite and affable man in the surgical instrument line, has an interesting advertisement in this journal. He has also doctor's saddle-bags, drug cases, etc. Go and see him.

THE SCIENTIFIC AMERICAN, published by the great patent agency firm of Munn & Co., New York, is the most practically useful publication of its kind in the country. Indeed, it occupies a field distinctively its own. Not alone for the machinist, manufacturer, or scientist, but it is a journal for popular perusal and study. It is the standard authority on scientific and mechanical subjects. It is placed at a very low rate of subscription, $3 per annum, which places it within the reach of all.

TREATMENT OF TYPHOID FEVER.—The Indiana *Medical Journal* summarises the most successful treatment yet discovered for typhoid fever in the following words: "Milk and animal broths, with quinine, antipyrin, and cold bathing, and the judicious use of stimulants" So far as our experience goes, even this short array of agencies employed may be curtailed, in many cases, by the leaving off of the milk and broths, as the patient cannot or will not take them until the fever begins to subside.

WE have just received some neat little prescription and memorandum books issued by the Mellier Drug Company, of St. Louis, dealers in physician's supplies, surgical instruments and appliances of all kinds, and proprietors of Tongaline, Mellier's standard—Elliott patent—saddle-bags and buggy-cases, and Mellier's improved uterine supporters. The book contains a very convenient table for approximating at a glance date of confinement, when date of last menstruation is given. It will be mailed to any physician, who will send his address to the Mellier Drug Company, St. Louis, and mention this journal.

LACTOPEPTINE PREPARATIONS.—We are in receipt from the New York Pharmacal Association of beautiful samples of the following excellent preparations: *lactopeptine powder*, *lactopeptine syrup phosphates*, *lactopeptine*

*liquid*, and *lactopeptine elixir*. These preparations are prepared with great care, and put up in most elegant style, and are recommended as superior to pepsin in digestive ailments, in summer complaints of children, and in many disorders of the assimilative organs. See the advertisement of New York Pharmical Association in this journal.

